# Metal- and Oxidant-Free Photoinduced Aromatic Trifluoromethylation Performed in Aerated Gel Media: Determining the Effects on Yield and Selectivity

**DOI:** 10.3390/molecules24010029

**Published:** 2018-12-21

**Authors:** Alex Abramov, Hendrik Vernickel, César Saldías, David Díaz Díaz

**Affiliations:** 1 Institut für Organische Chemie, Universität Regensburg, Universitätsstr. 31, Regensburg 93053, Germany; Alex.Abramov@chemie.uni-regensburg.de (A.A.); Hendrik.Vernickel@stud.uni-regensburg.de (H.V.); 2 Departamento de Química Física, Facultad de Química, Pontificia Universidad Católica de Chile, Casilla 302, Santiago Correo 22, Chile; casaldia@uc.cl; 3 Instituto de Productos Naturales y Agrobiología del CSIC, Avda. Astrofísico Francisco Sánchez 3, 38206 La Laguna, Tenerife, Spain

**Keywords:** trifluoromethylation, photoinduced reaction, chemoselectivity, supramolecular gel, confined reaction media

## Abstract

In this work we have investigated the potential benefits of using supramolecular gel networks as reaction media to carry out air-sensitive metal-free light-induced trifluoromethylation of six-membered (hetero)arenes under aerobic conditions. This reaction was performed at room temperature (RT) using sodium triflinate (CF_3_SO_2_Na, Langlois’ reagent) as a source of radicals and diacetyl as electron donor. The effects of confinement in gel media, concentration of reactants, and type of light source on yield and product distribution were evaluated and compared to the results obtained in homogeneous solution. Four different low molecular weight (LMW) gelators were employed in this study. The results confirmed the blocking effect of the gel medium against reaction quenching by external oxygen, as well as a certain control on the kinetics and selectivity.

## 1. Introduction

Millions of years of natural evolution have developed compartmentalized multifunctional systems such as cell organelles to facilitate challenged chemical reactions under very mild conditions and with a precise control on kinetics and selectivity. This has inspired many research teams all over the world to fabricate numerous nano- and microreactors based on the self-assembly of low-molecular-weight (LMW) molecules via non-covalent interactions (e.g., hydrogen-bonding, π-π stacking, van der Waals, charge-transfer,) [1,2]. In particular, the field of photochemistry has been a big beneficiary of these materials [3,4], where key aspects that control photoinduced processes, such as light absorption and lifetime of redox intermediates, are improved in confined environments including, for example, mesoporous inorganic materials [5], microemulsions [6], micelles [7,8], vesicles [9], foams [10], polyelectrolyte nanoparticles [11], and gels [12,13,14]. Among these systems, supramolecular or physical gels are viscoelastic materials made of LMW compounds (i.e., gelators) that self-assembly into 3D networks through non-covalent interactions [15]. In general, the formation of these gels involves first the self-assembly of gelator molecules into 1D fibers of nm diameter and mm length followed by their physical entanglement. The solid-like aspect of the gels results from the immobilization of solvent molecules (major component) into the interstices of the 3D network via surface tension and capillary forces, increasing the viscosity of the medium by factors up to 10^10^. Due to the non-covalent nature of the interactions, most supramolecular gels show reversible *gel*-to-*sol* transitions that can be triggered by different external stimuli (e.g., temperature, pH, ionic strength, irradiation) [15].

Recently, we have demonstrated the potential of physical gels as confined microenvironments to perform photochemical reactions under conditions milder than in solution [16,17]. Specifically, photoreduction of aryl halides by low-energy light irradiation of a suitable donor/acceptor pair embedded in a physical gel [16], as well as photoinduced C–C cross-coupling reactions (i.e., functionalization of aryl halides, trifluoromethylation of arenes) [17], were successfully carried out under aerobic conditions in spite of the high air-sensitivity of these processes. The results derived from these extensive studies suggest that triplet sensitized chemical reactions could take place either inside the gel nanofibers [16] and/or within the solvents pools compartmentalized in between the fibers [17], where the volume/surface ratio and viscosity changes dramatically compared to homogeneous solution. The exact location of photocatalytic species in the gel system seems to be influenced by the hydrophobic/hydrophilic balance between the photocatalyst and the surrounded medium (e.g., nanofibers, liquid). Thus, the combination of high viscosity and numerous liquid interfaces in physical gels constitute one of the key factors that facilitate oxygen-sensitive photochemical reactions in air. Other potential effects such as increased local substrate concentration and/or affinity of substrates and/or catalytic species to the surface of the fibers should also be taken into consideration. Furthermore, the impact of hydrogel and micellar media on the photodimerization of acenaphthylene has been studied by Maitra and co-workers [18]. Interestingly, the results showed that the *syn*-to-*anti* ratio was greater in gel-bound state compared to solution, being the selectivity apparently correlated with the rigidity of the gels [18].

In this work, we have expanded the study of photoinduced processes in supramolecular gel networks focusing on the metal-free light-induced trifluoromethylation of six-membered heteroarenes and arenes at room temperature (RT) using sodium triflinate (CF_3_SO_2_Na, Langlois’ reagent) as a source of radicals and diacetyl as electron donor. The effects of confinement in gel media, concentration of reactants, and type of light source on product distribution were evaluated and compared to the results obtained in homogeneous solution.

## 2. Results and Discussion

**Scheme 1:**
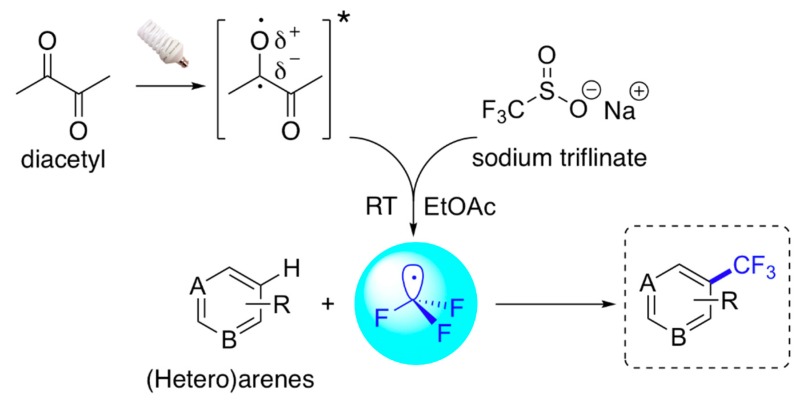
General scheme of photoinduced trifluoromethylation of arenes and heteroarenes upon light irradiation in the presence of sodium triflinate and diacetyl.

Recently, Li and co-workers [19] described an easy, metal- and oxidant-free photochemical strategy for the direct trifluoromethylation of unactivated arenes and heteroarenes under visible light irradiation using sodium triflinate (CF_3_SO_2_Na) as source of trifluoromethyl radicals (CF_3_^∙^) and aliphatic diacetyl as radical initiator (photosensitizer) (Scheme 1).

The process involves the initial excitation of diacetyl, which causes the transition of a lone pair electron from oxygen to the carbonyl carbon. Then, the resulting electron-deficient oxygen atom of the carbonyl n, p* state abstracts one electron from CF_3_SO_2_Na to generate electron deficient CF_3_^∙^ radicals, which further react with the most electron-rich position of the arene affording the corresponding trifluoromethylated arenes.

In general, this reaction constitutes an important process as the trifluoromethylated aromatic products are structures of high relevance in modern medicinal chemistry [20,21,22]. Importantly, due to the air-sensitivity of the excited reactive intermediates, the reaction is carried out in solution under inert atmosphere. Inspired by our previous investigations [16,17], we explored the feasibility of this process in supramolecular gel media, which should facilitate the reaction under aerobic conditions.

First of all, an initial set of control experiments were carried out using the photoinduced trifluoromethylation of 1,3,5-trimethoxybenzene (**1**) at RT in the presence of diacetyl as model reaction and using LEDs (λ_ex_ = 400 nm) as visible light source (Table 1). As starting point, we carried out the reactions using the amounts previously described [19]: arene (0.1 mmol), CF_3_SO_2_Na (0.4 mmol), in a mixture of ethyl acetate (0.8 mL)-diacetyl (0.2 mL, 2.1 mmol). The reaction performed in solution under vigorous stirring and degassing conditions afforded the corresponding monosubstituted and disubstituted trifluoromethylated compounds (**2** and **3**, respectively) in an overall yield of 96%, favoring the monosubstituted product (i.e., **2** = 79%, **3** = 17%) (Table 1, entry 1). As expected for an oxygen-sensitive process, when the reaction was performed without degassing the overall yield drastically dropped to 37% (i.e., **2** = 28%, **3** = 9%) (Table 1, entry 2). Interestingly, the reaction carried out in gel medium (using **G-1** as LMW gelator, *c* = 20 g L^−1^) under non-stirred and aerobic conditions afforded the desired products in an overall yield of 85% (i.e., **2** = 61%, **3** = 24%) (Table 1, entry 2). It is worth mentioning that the use of degassed gel media did not provide better results. Interestingly, the percentage ratio **2**/**3**, taking into consideration the batch-to-batch experimental error (±2%), was different in each case. Although the balance was always favored to the monosubstituted product **2**, the amount of disubstituted product **3** significantly increased when the reaction was carried out in gel phase compared to homogeneous solution. This preliminary observation pointed out a possible effect of the gel network on the chemoselectivity of the reaction, while maintaining a good conversion compared to the reaction under inert conditions.

It is important to emphasize that the gelator concentrations explored in this work were selected to ensure that the gel medium remains stable after doping with reactants, as well as during the experiment (e.g., no melting or phase separation). For example, the *gel*-to-*sol* transition temperature (*T*_gel_) of the undoped gel made of **G-1** (*c* = 20 g L^−1^, Table 1, entry 3) was 56 ± 2 °C. The incorporation of reactants into the gel caused a drop of the *T*_gel_ to 44 ± 2 °C, which still allowed the gel to remain stable for the experiments. 

The observed destabilization of the gel upon inclusion of external reactants is not surprising because they do not form part of the supramolecular gel network, which is a metastable state between solution and crystallization [23]. Moreover, the *T*_gel_ of the doped gel after irradiation underwent a minor increment to 50 ± 2 °C. These results suggested a good tolerance of the gel medium toward the reaction conditions, conversion of reactants, and reaction time.

**Figure 1:**
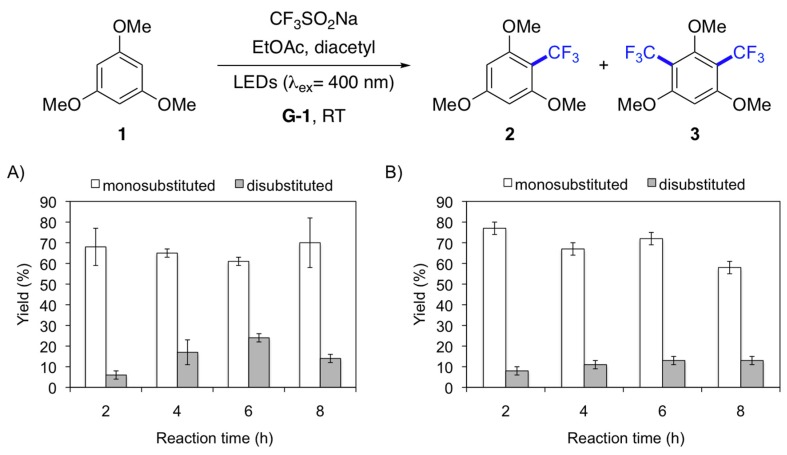

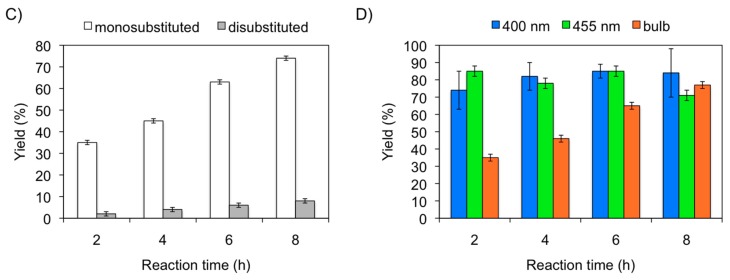
Effect of light source on the yield and product distribution of a trifluoromethylation model reaction in gel medium. Irradiation source: (**A**) LEDs (λ_ex_ = 400 nm); (**B**) LEDs (λ_ex_ = 455 nm); (**C**) CFL (bulb). Reaction conditions: 1,3,5-Trimethoxybenzene (16.8 mg, 0.1 mmol), sodium triflate (62.4 mg, 0.4 mmol), EtOAc (0.8 mL), diacetyl (0.2 mL), **G-1** (*c* = 20 g L^−1^), light irradiation, air, RT. (**D**) Overall yield (**2** + **3**) obtained with different irradiation sources. Yields were calculated by ^19^F-NMR using hexafluorobenzene (11.5 µL, 0.1 mmol) as internal standard. Monosubstituted product = **2**; disubstituted product = **3**.

At this point, we investigated the effect of the light source on the product distribution of the model reaction in gel medium (Figure 1). In this study, both the yields and product distribution was determined over time (i.e., 2 h, 4 h, 6 h, 8 h) using different light sources (i.e., LEDs: l_ex_ 400 nm, 455 nm, and compact fluorescent lamp (CFL, bulb)). The results showed that irradiation with LEDs at 400 nm afforded the monosubstituted product **2** in ca. 60% within 2 h, while the disubstituted compound **3** was obtained in ca. 4% (Figure 1A). Interestingly, increasing the reaction time to 6 h caused only a significant increase of **3** until ca. 24%. A similar constant production of monosubstituted compound **2** (ca. 70%) was observed upon irradiation with LEDs at 455 nm within 2–6 h (Figure 1B). However, the disubstituted derivative **3** remained almost constant (ca. 10%) during this period. In contrast to LEDs, the use of CFL (bulb) as light source provides a good degree of chemoselectivity, affording mainly the monosubstituted product **2**, whose yield gradually increased over time. Specifically, compound **2** was obtained in ca. 74% yield after 8 h, while only ca. 5% of the disubstituted derivative **3** was observed (Figure 1C). In general, neither longer reaction times nor irradiation below 400 nm provided further improvements (data not shown). Although LED irradiation displayed the fastest reaction kinetics and highest overall yield (i.e., **2** + **3**), the use of CFL (buld) enabled a more gradual increment of product yield and better chemoselectivity (Figure 1D). The differences observed between LED and CFL irradiation could be explained by the higher power output of LEDs, which generate a higher concentration of reactive radicals early in the reaction. On the other hand, CFL displays a broader wavelength distribution of the visible spectra, which results in the generation of less radicals leading predominantly to monosubstituted products.

Further experiments the feasibility of the model intragel reaction using different LMW gelators (Table 2). Polyamide-based gelators such as **G-1** and **G-2** provided high overall yields (>90%) at optimized gelator concentration (Table 2, entries 2, 5). In terms of product distribution, the monosubstituted compound **2** was the major product in both cases, albeit **G-1** offered higher chemoselectivity under optimized conditions. In contrast, the use of (di-Fmoc-lysine)-based gelator **G-3** gave lower overall yields, but with higher selectivity regardless the gelator concentration (i.e., monosubstituted product **2** was obtained almost exclusively) (Table 2, entries 7–9).

Finally, bis-urea **G-4** showed an intermediate situation in terms of overall yield and selectivity (Table 2, entry 10). However, further detailed studies are still necessary in order to establish proper structure-properties relationships. Additionally, a significant effect of the gelator concentration was also observed. An optimal gelator concentration was found for **G-1** and **G-2**, at which both the selectivity and yield were maximized (Table 2, entries 2, 5). A drop in selectivity and yield was observed above the optimal gelator concentration (Table 2, entries 3, 6). Thus, the use of **G-1** at a concentration of 25 g L^−1^ provided full conversion and the maximum amount of monosubstituted product **2** (93%), and a percentage ratio **2**:**3** > 13:1. Thus, these conditions were taken to carry out further studies. We hypothesized that below the optimal concentration there is a faster rate of oxygen diffusion, which facilitate the quenching of excited species causing a drop in overall yield. Moreover, above the optimal concentration the diffusion of reactants inside the solvent pools may be reduced causing a similar effect on the product yield. However, the effect of the gelator concentration on the selectivity remains unclear and further detailed experiments are still necessary to clarify this point.

**Figure 2:**
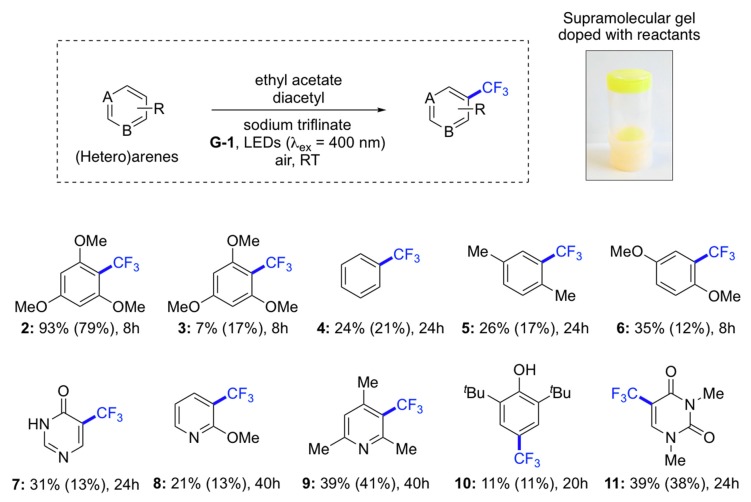

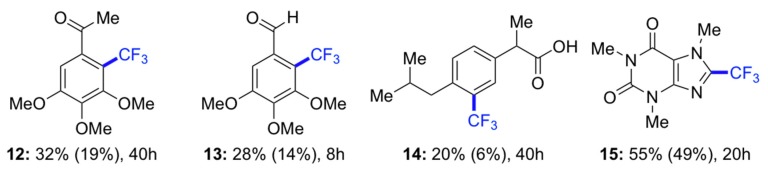
Substrate scope and relative comparison between solution and gel as reaction medium. Reaction conditions (unless otherwise indicated): (Hetero)arene (0.1 mmol), sodium triflinate (4 equiv), **G-1** (*c* = 25 g L^−1^), EtOAc (0.8 mL), diacetyl (0.2 mL), LEDs (λ_ex_ = 400 nm), 8–40 h, air, RT. Yields obtained in homogeneous solution are given in brackets. Yields were calculated by ^19^F-NMR using hexafluorobenzene (1 equiv) as internal standard. Average yields of at least two independent experiments are shown with a batch-to-batch estimated error of ±2%. Note: The use of other light sources and reactions conditions may alter the product yields.

A substrate scope study carried out within 8 h confirmed that the metal-free diacetyl-based trifluoromethylation requires highly electron-rich substrates (Figure 2). The best yields in gel phase under aerobic conditions were obtained with 1,3,5-trimethoxybenzene (**1**) (product **2**, 93%) and caffeine (product **15**, 55%). No disubstituted products were observed, except for the model reaction performed with **1** (vide supra). Very interestingly, the use of aerated gel medium afforded, in most cases, the desired products in higher yields compared to those obtained in degassed solution. It is important to emphasize that the comparisons between reactions in solution and in gel media were performed exactly under the same conditions in terms of reaction volume, time, temperature, light irradiation, and stoichiometry. These results are in very good agreement with our previous observations [16,17], and suggest a stabilizing effect on the reactive intermediates by the surrounding supramolecular network.

**Figure 3:**
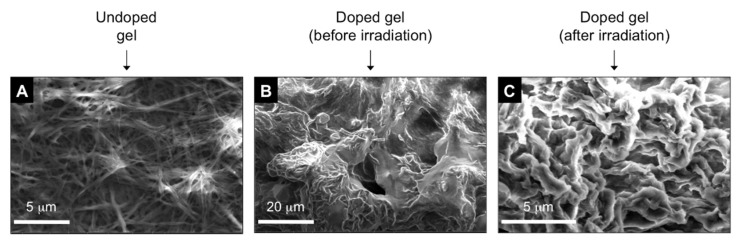
Representative scanning electron microscopy images of some xerogels prepared by freeze-drying the corresponding gels: (**A**) gel made of **G-1** (*c* = 25 g L^−1^) in EtOAc (0.8 mL); (**B,C**) gel made of **G-1** (*c* = 25 g L^−1^), and containing 1,3,5-trimethoxybenzene (16.8 mg, 0.1 mmol), sodium triflinate (62.4 mg, 0.4 mmol), EtOAc (0.8 mL), and diacetyl (0.2 mL).

Finally, comparative field-emission scanning electron microscopy (FE-SEM) indicated that the inclusion of reactants in the gel caused an apparent densification of the network, which conserved a fibrillar aspect (Figure 3A,B). The images of the reactive gel (i.e., gel doped with reactants) before and after irradiation showed similar morphological features (Figure 3B,C). In agreement with our previous observations [17], this suggests that the sensitized chemical reaction occurs most likely in the solvent pools held between the fibers. The compartmentalized nature of the gel medium, which increases the surface-to-volume ratio compared to solution, together with a potential increment of local substrate concentration, and reduction of the diffusion of external oxygen through the viscous gel/air interface, seem to play a crucial role for the occurrence of the photoinduced process under aerobic conditions, with similar reaction kinetics [13], and higher product selectivity compared to homogeneous solution.

## 3. Conclusions

In conclusion, we have demonstrated that air-sensitive photoinduced trifluoromethylation of (hetero)arenes using Langlois’ reagent and diacetyl can be performed under aerobic conditions using supramolecular gels as reaction media. Importantly, different variables have been found to influence the yields and selectivity of this process, including principally the type of gelator and its concentration, as well as the light source. In most cases, under comparable conditions, the reaction with different substrates performed in aerated gels afforded the desired products in higher yields than in degassed solutions. The use of physical gels as reaction media may also be useful in high-throughput studies to optimize the conditions and catalysts for air-sensitive photochemical reactions.

## 4. Materials and Methods

### 4.1. Materials

Unless otherwise specified, all reagents, starting materials and solvents (p.a. grade) were purchased from commercial suppliers, i.e., ABCR (Karlsruhe, Germany), Acros (Geel, Belgium), Sigma-Aldrich (St. Louis, MO, USA), TCI (Tokyo, Japan) or Merck (Kenilworth, NJ, USA) and used as received without further purification. Reactions with oxygen- or moisture-sensitive reagents requiring oxygen- or moisture-sensitive reagents were carried out using flame-dried glassware, degassed solvents and Schlenk lines.

### 4.2. Synthesis and Characterization of Compounds

#### 4.2.1. General Remarks

Nuclear magnetic resonance (NMR) spectra were recorded with an Avance 400 (^1^H-NMR: 400 MHz, ^19^F-NMR: 376 MHz) or an Avance 300 (^1^H-NMR: 300 MHz, ^19^F-NMR: 282 MHz) instrument (Bruker, Billerica, MA, USA). Chemical shifts (δ) are reported in parts per million (ppm) relative to residual solvent peak (^1^H-NMR: CHCl_3_ = 7.26 ppm). Coupling constants (*J*) are given Hertz (Hz). The following notations are used to indicate the multiplicity of the signals: s = singlet, d = doublet, m = multiplet. High-resolution mass spectra (HRMS) were obtained according to the IUPAC recommendations (2013) from the central analytic mass spectrometry facilities of the Faculty of Chemistry and Pharmacy at the University of Regensburg. Column chromatography and flash chromatography were performed using silica gel with particle size 63–200 µm and 40–63 µm, respectively, as the stationary phase. Thin layer chromatography (TLC) analyses were performed using pre-coated TLC-sheets ALUGRAM^®^ Xtra SIL G/UV_254_ (Macherey-Nagel GmbH & Co. KG, Düren, Germany). Visualization was accomplished with UV light (λ_max_ = 365 nm). UV-Vis measurements were performed on a DH-2000-BAL UV-VIS-NIR spectrophotometer (Ocean Optics, Dunedin, FL, USA).

Photochemical reactions were performed using a custom-made set-up [13] with an array of suitable LEDs (3.5 V, 700 mA), i.e., λ_ex_ = 455 ± 15 nm, connected to a power supply. The irradiation device was equipped with a stainless-steel jacket to maintain refrigeration of the vials. The distance between the LEDs and the reaction vials was adjusted to 0.9 ± 0.1 cm. The apparatus also allows magnetic stirring of the reaction mixtures. The reported yields are referred to the isolated compounds unless otherwise stated. Oxygen- and moisture-free reactions were carried out with dry and degassed solvents, as well as glassware subjected to several evacuation (vacuum)/refill (nitrogen) cycles. Reactions with bulb light were performed using a Megaman bulb (Compact 2000 HPF, 30 Watt, 145 mA, 6500 K, 220–240) placed inside a custom-made chamber. The reaction vials were placed in a crystallizing dish containing cooling water. The bulb was located below the crystallizing dish (ca. 10 cm).

#### 4.2.2. Synthetic Procedures and Characterization of Compounds

Photoinduced trifluoromethylation reactions described under degassing conditions were performed under nitrogen atmosphere in a Schlenk line after freeze-pump-thaw cycles (4×) [19]. Gelators *N,N’*-*bis*-(octadecyl)-L-Boc-glutamic diamide (**G-1**) [24], *N,N’*-((1*R*,2*R*)-cyclohexane-1,2-diyl)didodecanamide (**G-2**) [25], and 1,1’-(4-methyl-1,3-phenylene)bis(3-(2-ethylhexyl)urea) (**G-4**) [26], were synthesized as we have previously described [17]. Gelator *Nα,Nε*-di-Fmoc-L-lysine (**G-3**) [27] was purchased from TCI Europe (catalog number B4931, CAS number 78081-87-5) and used as received.

### 4.3. Preparation and Characterization of Gel Materials

#### 4.3.1. General Procedure for Gel Preparation

Control gels (i.e., gels without reactants) were prepared in 5 mL snap glass vials having a specific amount of the desired gelator and solvent (p.a. grade). The mixture was gently heated with a heat gun until the solid material was completely dissolved. The resulting isotropic solution was allowed to cool down to RT affording the corresponding gels. No control over temperature rate during the heating-cooling process was applied. In general, the formation of reactive gels (i.e., gels with reactants) was achieved after ultrasound-assisted homogenization of the mixture containing gelator, solvent and reactants, gently heating of the mixture with a heat gun and final cooling down to RT (vide infra).

The lowest gelator concentration used in each case was selected based on both the stability of the gel medium after doping with reactants and the stability during the experiment (e.g., no melting or phase separation).

*Gel*-to-*sol* transition temperatures (*T*_gel_) were determined using an oil bath placed on an electric heating plate equipped with a temperature control couple (heating rate of ca. 1 °C/min). The temperature at which the gel started to break was defined as *T*_gel_ with an estimated error of ±2 °C after several heating-cooling cycles. Each measurement was made at least by duplicate and the average value reported.

The morphological characterization of the samples was performed using an FEI QUANTA FEG 250 field emission scanning electron microscope (Thermo Fisher Scientific, Waltham, MA, USA). The electron micrographs were recorded in high-vacuum mode under an acceleration voltage of 5 kV. The preparation of samples was carried out as follows: using a microsyringe a determined sample volume from an ethanol solution (1 g L^−1^) was taken and deposited onto a silicon wafer substrate. After this, evaporation of the solvent was carried out under vacuum at RT for 24 h. Finally, the samples were lyophilized prior visualization. Images were taken at the Pontificia Universidad Católica de Chile (Centro de Investigación en Nanotecnología y Materiales Avanzados).

#### 4.3.2. General Procedure for Intragel Reactions and Product Analysis

An oven-dried 5 mL vial was loaded with sodium triflate (62.4 mg, 0.4 mmol), the desired arene (0.1 mmol) and **G-1** gelator (25.0 g L^−1^). The vial was equipped with a septum through which EtOAc (0.8 mL) and diacetyl (0.2 mL) were added with a syringe. The resulting mixture was homogenized in an ultrasonic bath (USC200TH, 45 kHz, 120 Watt, VWR™, Leicestershire, England) at constant temperature (23 ± 2 °C) for ca. 1 min and heated with a heat gun (heating level 3 of 10) until the gelator was completely dissolved. The gel was formed during sonication or after cooling down the mixture to RT. Then, the vials were placed under LED (l = 400 nm or 455 nm, as indicated) light irradiation during the specified time (vide infra). Subsequently, the vials were heated up with a heat gun (heating level 3 of 10) until dissolution of the gel phase. At this point, EtOAc (1.0 mL) and hexafluorobenzene (11.6 µL, 0.1 mmol, NMR internal standard) were added into the hot solution and 5 drops of the crude solution were taken out for NMR analysis. Notes: (1) Reactions using different gelators, light source, gelator concentration and/or reactants concentration were performed following the same procedure using the amounts given in the main text. (2) Gelator **G-3** required 5 min sonication for complete dissolution in the reaction mixture, and gelation occurred during this process.

The above procedure was applied for the intragel synthesis of compounds **1**–**13**. Starting material (SM), amount of SM and reaction time are given for each case. Although some representative characterization data such as the distinctive ^19^F-NMR peaks are included below, all products described in this paper has been previously described in the cited references.

*1,3,5-Trimethoxy-2-(trifluoromethyl)benzene* (**2**, *monosubstituted*; **3**, *disubstituted*) [19]: SM = 1,3,5-trimethoxybenzene (16.8 mg, 0.1 mmol). After 8 h, 3 mL of brine was given to the reaction mixture and it was extracted using 2 mL EtOAc (3×). The combined organic mixtures were dried over Na_2_SO_4_ and purified by chromatography using 5–15% ethyl acetate in petroleum ether to provide the title compound **2** (14.0 mg, 59% yield) as a white solid powder and byproduct **3** (3.0 mg, 10% yield) as a white solid powder.

**2**: ^1^H-NMR (CDCl3) δ 3.84 (s, 9H, CH_3_); 6.13 (s, 2H, ArH) ppm; ^19^F-NMR (CDCl_3_) δ −54.63 ppm. **3**: ^1^H-NMR (CDCl3) δ 3.82 (s, 3H, CH_3_); 3.93 (s, 6H, CH_3_), 6.35 (s, 1H, ArH) ppm; ^19^F-NMR (CDCl_3_) δ −55.99 ppm. TLC in 5% EtOAc in petroleum ether: 1,3,5-trimethoxybenzene, R_f_ = 0.56; **2**, R_f_ = 0.27; **3**, R_f_ = 0.17.

*Trifluoromethylbenzene* (**4**) [28]: SM = benzene (9 µL, 0.1 mmol). Reaction time = 24 h: ^1^H-NMR (CDCl_3_) δ 7.34–7.42 (m, 2H, ArH); 7.45 (d, 1H, ArH, *J* = 7.1 Hz), 7.51 (d, 2H, ArH, *J* = 8.1 Hz) ppm; ^19^F-NMR (CDCl_3_) δ –63.30 ppm.

*1,4-Dimethyl-2-(trifluoromethyl)benzene* (**5**) [28]: SM = *p*-xylene (13 µL, 0.1 mmol). Reaction time = 24 h. ^1^H-NMR (CDCl_3_) δ 2.26 (s, 3H, CH_3_); 2.33 (s, 3H, CH_3_); 7.05 (d, 1H, ArH, *J* = 7.7 Hz); 7.11 (d, 1H, ArH, *J* = 8.2 Hz), 7.28 (s, 1H) ppm; ^19^F-NMR (CDCl_3_) δ –62.3 ppm.

*1,4-Dimethoxy-2-(trifluoromethyl)benzene* (**6**) [28]: SM = 1,4-dimethoxybenzene (14.0 mg, 0.1 mmol). Reaction time = 8 h; ^1^H-NMR (CDCl_3_) δ 3.68 (s, 1H, CH_3_); 3.75 (s, 1H, CH_3_); 6.86 (d, 1H, ArH, *J* = 9.2 Hz); 6.92 (dd, 1H, ArH, *J* = 9.2, 3.2 Hz), 6.98 (d, 1H, ArH, *J* = 3.0 Hz) ppm; ^19^F-NMR (CDCl_3_) δ −63.03 ppm.

*5-(Trifluoromethyl)pyrimidin-4(3H)-one* (**7**) [28]: SM = 4(3*H*)-pyrimidinone (9.6 mg, 0.1 mmol). Reaction time = 24 h; ^19^F-NMR (CDCl_3_) δ −66.15 ppm.

*2-Methoxy-3-(trifluoromethyl)pyridine* (**8**) [28]: SM = 2-methoxypyridine (10.5 µL, 0.1 mmol). Reaction time = 40 h; ^19^F-NMR (CDCl_3_) δ −64.59 ppm.

*2,4,6-Trimethyl-3-(trifluoromethyl)pyridine* (**9**) [28]: SM = 2,4,6-trimethylpyridine (13.5 µL, 0.1 mmol). Reaction time = 40 h; ^19^F-NMR (CDCl_3_) δ −54.92 ppm.

*2,6-Di-tert-butyl-4-(trifluoromethyl)phenol* (**10**) [19]: SM = 2,6-di-*tert*-butylphenol (20.6 mg, 0.1 mmol). Reaction time = 20 h; ^9^F-NMR (CDCl_3_) δ −61.81 ppm.

*1,3-dimethyluracil* (**11**) [28]: SM = (14.0 mg, 0.1 mmol). After 24 h, 3 mL of brine was given to the reaction mixture and it was extracted using EtOAc (3×) and CH_2_Cl_2_ (1×). The combined organic mixtures were dried over anhydrous Na_2_SO_4_ and purified by chromatography using 10–60% EtOAc in petroleum ether to provide the title compound (6 mg, 30% yield) as a yellowish solid powder. ^1^H-NMR (CDCl3) δ 3.36 (s, 3H, CH_3_); 3.49 (s, 3H, CH_3_), 7.67 (d, 1H, *J* = 1.0 Hz) ppm; ^19^F-NMR (CDCl_3_) δ −64.3 (s) ppm.

*3,4,5-Trimethoxy-2-(trifluoromethyl)acetophenone* (**12**) [19]: SM = 3,4,5-trimethoxyacetophenone (21 mg, 0.1 mmol). Reaction time = 40 h; ^19^F-NMR (CDCl_3_) δ −55.64 ppm.

*3,4,5-Trimethoxy-2-(trifluoromethyl)benzaldehyde* (**13**) [19]: SM = 3,4,5-trimethoxybenzaldehyde (19.6 mg, 0.1 mmol). Reaction time = 8 h; ^19^F-NMR (CDCl_3_) δ −51.74 ppm.

*2-(4-Isobutyl-3-(trifluoromethyl)phenyl)propanoic acid (CF_3_-ibuprofen)* (**14**) [28]: SM = ibuprofen (20.8 mg, 0.1 mmol). Reaction time = 40 h; ^19^F-NMR (CDCl_3_) δ −59.52 ppm.

*CF_3_-Theobromine (CF_3_-caffeine)* (**15**) [19]: SM = caffeine (19.4 mg, 0.1 mmol). Reaction time = 20 h; ^19^F-NMR (CDCl_3_) δ −63.13 ppm.

## Figures and Tables

**Table 1:** Preliminary control trifluoromethylation reactions in solution and gel media. ^1^

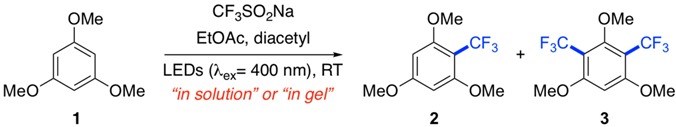				
Entry	Reaction Medium	Yield 2 (%) ^3^	Yield 3 (%) ^3^	Yield 2 + 3 (%) ^3^
1	Solution, degassing	79	17	96
2	Solution, no degassing	28	9	37
3	Gel ^2^, no degassing	61	24	85

^1^ Reaction conditions (unless otherwise indicated): 1,3,5-Trimethoxybenzene (16.8 mg, 0.1 mmol), sodium triflinate (62.4 mg, 0.4 mmol), EtOAc (0.8 mL), diacetyl (0.2 mL), LEDs (λ_ex_ = 400 nm), RT, 6 h. Reactions in solution were carried out in stirred vessels. ^2^ **G-1** (*c* = 20 g L^−1^) was used as LMW gelator. ^3^ Yields were calculated by ^19^F-NMR using hexafluorobenzene (11.5 µL, 0.1 mmol) as internal standard. Batch-to-batch estimated error of ±2%.

**Table 2:** Influence of LMW gelator and gelator concentration on the yield and product distribution of trifluoromethylation model reaction. ^1.^

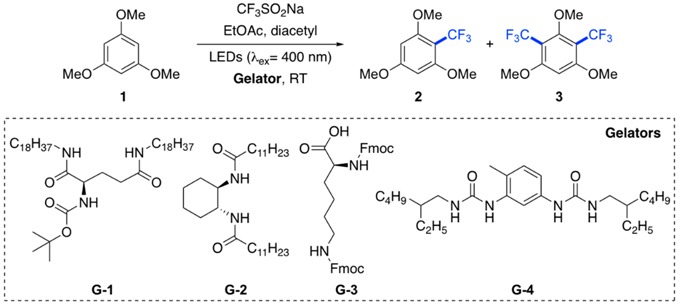					
Entry	Gelator	Concentration (g L^−1^)	Yield 2 (%) ^3^	Yield 3 (%) ^3^	Yield 2 + 3 (%) ^3^
1	G-1	20	61	24	85
2	G-1	25	93	7	100
3	G-1	30	73	19	92
4	G-2	25	72	11	83
5	G-2	30	79	14	93
6	G-2	35	68	8	76
7	G-3	60	52	2	54
8	G-3	65	55	2	57
9	G-3	70	50	2	52
10 ^2^	G-4	50	72	7	79

^1^ Reaction conditions (unless otherwise indicated): 1,3,5-Trimethoxybenzene (16.8 mg, 0.1 mmol), sodium triflate (62.4 mg, 0.4 mmol), EtOAc (0.8 mL), diacetyl (0.2 mL), LEDs (λ_ex_ = 400 nm), LMW gelator, 8 h, air, RT. ^2^ Reaction time = 6 h. ^2^ Yields were calculated by ^19^F-NMR using hexafluorobenzene (11.5 µL, 0.1 mmol) as internal standard. Average yields of at least two independent experiments are shown with a batch-to-batch estimated error of ±2%.
